# Metagenomic Analysis Reveals Changes in Bacterial Communities and Antibiotic Resistance Genes in an Eye Specialty Hospital and a General Hospital Before and After Wastewater Treatment

**DOI:** 10.3389/fmicb.2022.848167

**Published:** 2022-05-19

**Authors:** Xueli Ma, Xu Dong, Jiabei Cai, Chunyan Fu, Jing Yang, Yuan Liu, Yan Zhang, Tian Wan, Shudan Lin, Yongliang Lou, Meiqin Zheng

**Affiliations:** ^1^Eye Hospital and School of Ophthalmology and Optometry, Wenzhou Medical University, Wenzhou, China; ^2^National Clinical Research Center for Ocular Diseases, Wenzhou, China; ^3^Wenzhou Key Laboratory of Sanitary Microbiology, Key Laboratory of Laboratory Medicine, Ministry of Education, School of Laboratory Medicine and Life Sciences, Wenzhou Medical University, Wenzhou, China

**Keywords:** hospital wastewater, wastewater treatment, antibiotic resistance genes, bacterial communities, metagenomic

## Abstract

The spread of antibiotic resistant bacteria (ARB) and antibiotic resistance genes (ARGs) in hospital wastewater poses a great threat to public health, and wastewater treatment plants (WWTPs) play an important role in reducing the levels of ARB and ARGs. In this study, high-throughput metagenomic sequencing was used to analyze the bacterial community composition and ARGs in two hospitals exposed to different antibiotic use conditions (an eye specialty hospital and a general hospital) before and after wastewater treatment. The results showed that there were various potential pathogenic bacteria in the hospital wastewater, and the abundance and diversity of the influent ARGs in the general hospital were higher than those in the eye hospital. The influent of the eye hospital was mainly composed of *Thauera* and *Pseudomonas*, and *sul1* (sulfonamide) was the most abundant ARG. The influent of the general hospital contained mainly *Aeromonas* and *Acinetobacter*, and *tet39* (tetracycline) was the most abundant ARG. Furthermore, co-occurrence network analysis showed that the main bacteria carrying ARGs in hospital wastewater varied with hospital type; the same bacteria in wastewater from different hospitals could carry different ARGs, and the same ARG could also be carried by different bacteria. The changes in the bacterial community and ARG abundance in the effluent from the two hospitals showed that the activated sludge treatment and the direct chlorination disinfection can effectively remove some bacteria and ARGs in wastewater but have limitations. The species diversity increased significantly after the activated sludge treatment, while the direct chlorination disinfection did not increase the diversity. The activated sludge treatment has a better effect on the elimination of ARGs than the direct chlorination disinfection. In summary, we investigated the differences in bacterial communities and ARGs in wastewater from two hospitals exposed to different antibiotic usage conditions, evaluated the effects of different wastewater treatment methods on the bacterial communities and ARGs in hospital wastewater, and recommended appropriate methods for certain clinical environments.

## Introduction

The number of clinical pathogens with resistance against antibiotics is increasing rapidly, seriously endangering the sustainability of human and veterinary antibiotic use (Pruden et al., 2013). Wastewater treatment plants (WWTPs) are considered to be one of the hot spots for the release of antibiotic resistance genes (ARGs) into the environment. Some ARGs persist after treatment and can be released into natural aquatic systems (Michael et al., 2013); these genes have been detected in different environments, such as drinking water (Ma et al., 2019), lakes (Liu et al., 2018), rivers (Xia et al., 2017), and soil (Chen et al., 2016). Compared with urban and community wastewater, hospital wastewater may contain more ARGs. Hospital wastewater may contain pathogenic bacteria and high concentrations of antibiotics and other drugs (Baquero et al., 2008; Rodriguez-Mozaz et al., 2015). Bacteria isolated from hospital wastewater environments usually have multidrug resistance (MDR) (Manoharan et al., 2021); these resistance genes can be transmitted to the environment, and individual treatment of hospital wastewater has been considered as a supplement to urban wastewater treatment (Szekeres et al., 2017). Identifying the bacterial community composition and the abundance of ARGs in hospital wastewater is a prerequisite for choosing appropriate wastewater treatment methods to prevent ARGs from spreading to the environment.

The spread of ARGs is a serious threat to human health, wastewater treatment plays a vital role in eliminating ARB and ARGs. Several researchers are devoted to building new wastewater treatment technologies. To date, several strategies have been developed to minimize the harm of pollutants in wastewater, including oxidation (Ganiyu et al., 2021), biological treatment (Zhu et al., 2021), adsorption (Burakov et al., 2018), membrane bioreactor (Ryu et al., 2021), etc. However, these wastewater treatment techniques are designed to eliminate overall ARG levels, ignoring they can also create conditions suitable for the growth of antibiotic resistant bacteria (ARB) carrying certain ARGs (Moreira et al., 2018; Shi et al., 2021), and these surviving ARBs are harmful and may participate in horizontal gene transfer (HGT) between bacterial pathogens to facilitate the spread of ARGs (Makowska et al., 2020). To promote the faster development of wastewater treatment technology and make this strategy more effective, it is important to focus on the bacteria carrying ARGs that persist after wastewater treatment while eliminating overall ARGs levels.

Previous studies have shown high-level beta-lactam resistance genes are frequently found in hospital wastewater, and the abundance of which is related to antibiotic use and antibiotic residues in hospitals (Rowe et al., 2017; Rodriguez-Mozaz et al., 2020). However, bacterial community and the ARGs prevalence in different types of hospital wastewater were expected to be affected. Studies have shown that antibiotic usage varies among different diseases and infection sites, so the types and concentrations of antibiotics in wastewater from different hospitals are different (Coutu et al., 2013; Szekeres et al., 2017). The disease types associated with general hospitals are complex, and the antibiotics used are diverse, but the types of diseases treated in eye hospitals are relatively limited, and chloramphenicol, gentamicin, tobramycin, ciprofloxacin, and ofloxacin are the main antibiotics used to treat eye diseases (Watson et al., 2018). A comprehensive understanding of the distribution and function of ARB and ARGs in different types of hospitals wastewater is required to reduce the spread of ARGs.

The research on hospital wastewater is one of the hotspots in the field of wastewater. Several studies have shown hospitals wastewater contributed high concentrations of antibiotic resistance (AR) (Lamba et al., 2017; Szekeres et al., 2017), and a correlation between antibiotics, ARGs, and ARB in hospital wastewater have been found (Cai et al., 2021; Manoharan et al., 2021). Fluoroquinolones were detected at the highest concentration in hospital wastewater samples and the incomplete removal of antibiotics and ARGs in WWTP severely affected the receiving river (Rodriguez-Mozaz et al., 2015). In this study, we selected wastewater from an eye hospital and a general hospital before and after treatment and performed metagenomics analysis on them. We identified the distribution and prevalence of bacterial community and ARGs in the Eye and General hospital wastewater. That is significant for ensuring that appropriate treatment methods are capable to be used in certain clinical settings to prevent the ARGs in hospital wastewater from spreading into the environment and threatening the health of humans and animals. In addition, we attempted to evaluate the effects of the activated sludge treatment process and the direct chlorination disinfection treatment process on the bacterial communities and ARGs in hospital wastewater. This work may help promote the efficient development of wastewater treatment technology.

## Materials and Methods

### Sample Collection and DNA Extraction

**FIGURE 1:**
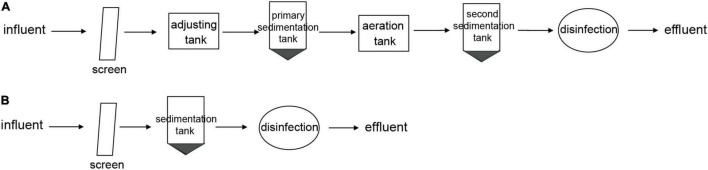
Wastewater treatment process of two hospitals. **(A)** Activated sludge wastewater treatment process in eye hospital. **(B)** Direct chlorination disinfection wastewater treatment process in general hospital.

Two hospitals (an eye specialty hospital and a general hospital in Zhejiang, China) were chosen for analysis. Influent and effluent samples were collected from the eye hospital and the general hospital before and after treatment. An activated sludge treatment process was applied in the eye hospital, and a direct chlorination disinfection treatment process was applied in the general hospital. Detailed information on the wastewater treatment processes of the two hospitals is provided in Figure 1.

Grab wastewater samples were collected from the raw influent (E.RW1-3 and G.RW1-3) and the final effluent (E.TW4-6 and G.TW4-6) of the eye hospital and general hospital. The samples were collected in sterilized Nalgene 1-liter polycarbonate bottles, transported to the laboratory in refrigerated containers, and processed within 12 h. To helps account for temporal variation, the sampling processes were repeated three times on April 16, April 27, and May 16 in 2021, and finally, a total of 12 samples were obtained. The volumes of wastewater filtered represented a compromise between the expected DNA extraction yield, DNA requirement, and filtration capacity of the membrane before collapse (Narciso-da-Rocha et al., 2018), that is, 200 mL of influent from the two hospitals, 500 mL of effluent from the eye hospital, and 300 mL of effluent from the general hospital, which were pre-filtered through 5-μm pore size polycarbonate filters to remove impurities. Then, each filtrate was filtered again using a 0.22 μm membrane (Whatman) filter to capture and concentrate microbes. The filter membranes were stored at −80°C until DNA extraction using the Powerwater DNA Kit (Qiagen, Hilden, Germany) according to the manufacturer indications. The concentration and quality of the extracted DNA were measured by spectrophotometry (DeNovix DS-11, United States). The degree of DNA degradation and potential contamination were examined by 1% agarose gel electrophoresis.

### Metagenomic Analysis

The genomic DNA was sequenced by the Illumina NovaSeq-6000 platform. All data were uploaded to the NCBI database under BioProject accession number PRJNA770854. KneadData was applied to the raw paired-end sequences to trim adaptors and filter low-quality reads (length < 50 bp, quality value < 20, or containing N bases).^1^ The remaining high-quality sequences were used for further analysis. Taxonomic classification at the species level was performed using Kraken2 (Wood et al., 2019).

The detection and quantification of the resistance profiles of metagenomic data were conducted using ARGs-OAP with the default parameters (Yang et al., 2016). The core database of ARGs-OAP was the Structured Antibiotic Resistance Genes (SARG) database, which includes 24 ARG types (e.g., tetracycline resistance genes) and 1,244 ARG subtypes (e.g., *tetA* and *tetB*). The classification of the mechanism categories of the ARGs detected was performed by mapping resistance subtypes into the CARD database (Alcock et al., 2020). 219,795,608 (40.42%) of the genes in the Kraken2 could be uniquely and reliably assigned to a phylum, 157,896,430 (29.03%) to a genus, and 134,542,775 (24.74%) to a species. Still, nearly half of the genes belonged to uncharacterized “microbial dark matter” (Oh et al., 2014).

### Statistical Analysis

Alpha and beta diversity index determination at the genus level, principal component analysis (PCA), and Procrustes analysis were conducted using the vegan package in R 4.0.5 software.^2^ The heatmap of ARGs and stacking diagrams of genera and ARGs were generated using the complex heatmap and ggalluvial packages, respectively. The co-occurrence patterns between ARG subtypes and microbial taxa were determined using network analysis. Spearman’s rank correlation coefficients (Spearman’s ρ > 0.8, *P* < 0.01) were calculated using Hmisc packages, and the network was visualized using the Gephi platform (Li et al., 2015). Significant differences in the relative abundance of bacterial groups and ARG subtypes at different taxonomic levels were identified using a two-sided Welch’s *t*-test *via* Statistics Analysis Metagenomic Profiles (STAMP) software v2.1.3 (Parks et al., 2014), and *P* < 0.05 was considered to be statistically significant.

## Results

### Microbial Community Diversity and Richness

**FIGURE 2:**
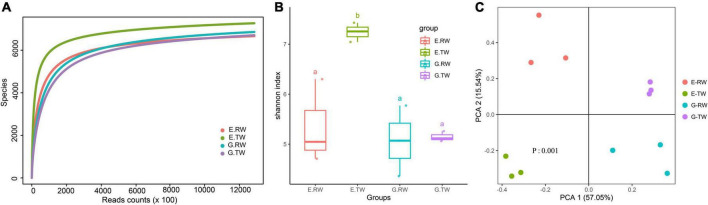
Alpha and beta diversity of microbial community. **(A)** Rarefaction curves of genus level. **(B)** Alpha diversity of four groups measured by Shannon diversity index. (a: No significant difference; b: significant difference) **(C)** Beta diversity of four groups measured by the PCA.

Rarefaction analysis showed that sequencing covered the largest species diversity in the four groups, implying that the sequencing depth of this study was sufficient (Figure 2A). Shannon’s diversity index and Simpson’s diversity index based on the genus profiles in the four groups were calculated to evaluate the community richness and diversity. The total number of reads obtained as well as the diversity indices are summarized in Table 1. The influent had lower microbial richness than the effluent, but the difference between the influent of the two hospitals was not significant. The Shannon index and Simpson index of the effluent of the eye hospital both increased significantly (*P* < 0.05), indicating that the activated sludge wastewater treatment process increased the abundance of microbial communities. However, the values for the general hospital, which used a direct chlorination disinfection treatment, did not increase (Figure 2B).

**TABLE 1:** List of samples used and overview of the total number of raw sequences and clean sequences, Shannon index, and Simpson index obtained for each library.

Locations	Samples	Raw reads	Clean reads	Shannon index	Simpson index
Eye hospital influent	E.RW.1	78,749,707	74,530,946	4.712	12.345
	E.RW.2	33,721,368	32,584,394	5.047	18.441
	E.RW.3	52,106,470	50,810,821	6.302	107.176
Eye hospital effluent	E.TW.4	43,906,451	41,883,561	7.044	152.788
	E.TW.5	39,886,889	37,964,624	7.256	177.738
	E.TW.6	44,369,148	43,286,885	7.428	364.500
General hospital influent	G.RW.1	49,439,600	47,368,291	4.362	20.015
	G.RW.2	40,211,581	39,218,420	5.070	34.970
	G.RW.3	41,047,141	39,921,259	5.770	29.217
General hospital effluent	G.TW.4	39,806,548	38,828,888	5.117	35.832
	G.TW.5	36,853,900	35,791,795	5.063	30.511
	G.TW.6	64,035,650	61,184,335	5.259	44.226

As shown in Figure 2C, PCA and PERMANOVA analysis showed that the influent and effluent from the two hospitals were clustered, and there were significant differences in beta diversity among the four groups (*P* = 0.001). Additionally, both the eye hospital and the general hospital showed stability in the effluent and high variability in the influent.

### Taxonomic Classification and Community Diversity

#### Bacterial Diversity at the Phylum Level

**FIGURE 3:**
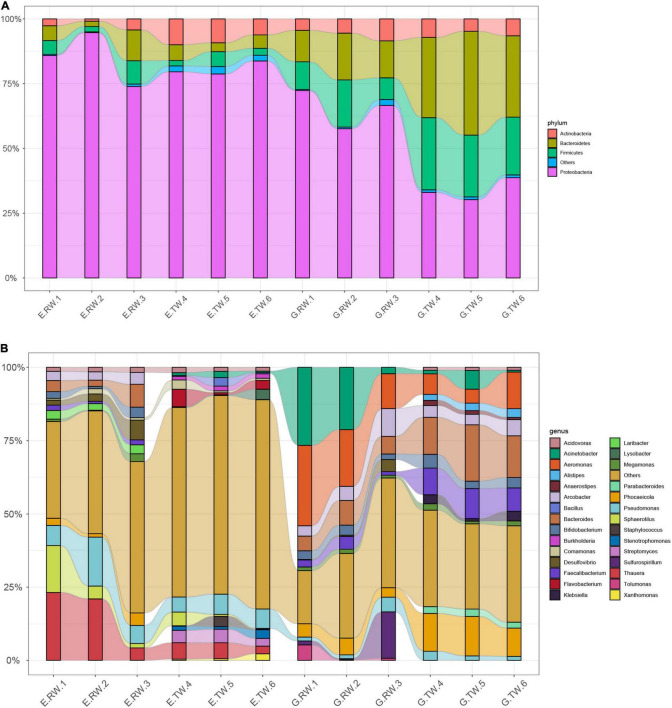
The relative abundance of bacteria **(A)** at the phylum level and **(B)** the genus level, less abundant (<1%) and unclassified taxa are grouped together as “others”.

The most abundant kingdom was bacteria, representing > 93% of the relative abundance in each individual sample, whereas the relative abundance of Archaea, Eukaryota, and Viruses were lower. The organisms from all kingdoms were classified into 39 phyla and 1423 genera. At the phylum level, the influent from the two hospitals were dominated by four phyla: *Proteobacteria* (E.RW 84.84%, G.RW 65.51%), *Bacteroidetes*, *Firmicutes*, and *Actinobacteria*, but the abundances were different (Figure 3A).

Among the effluent from eye hospital, the abundance of *Actinobacteria* increased (E.RW 2.60%, E.TW 8.45%, *P* < 0.05), but *Proteobacteria* (80.70%) was still the most abundant. The effluent from the general hospital showed that increased abundances of *Bacteroides* and *Firmicutes*, while the abundance of *Proteobacteria* (E.RW 65.51%, E.TW 33.99%, *P* < 0.05) decreased, and the relative abundance of the bacterial communities changed significantly (the relative abundances of *Bacteroides*, *Proteobacteria*, and *Firmicutes* were evenly distributed at approximately 30% each).

#### Bacterial Diversity at the Genus and Species Levels

At the genus level, *Thauera* (16.17%) and *Pseudomonas* (9.92%) were dominant in the eye hospital influent, and their abundances decreased to 4.62 and 6.19%, respectively, in the effluent, while *Thauera* and *Pseudomonas* accounted for only 0.09 and 1.69%, respectively, in the general hospital influent. Similarly, *Aeromonas* (19.57%) and *Acinetobacter* (16.65%) were dominant in the general hospital influent, and their abundances decreased to 8.05 and 2.75%, respectively, in the effluent, while the proportion of these two bacteria in the influent of the eye hospital was less than 1% (Figure 3B).

Further analysis at the species level showed that the most abundant *Pseudomonas* species in the eye hospital influent were *Pseudomonas alcaligenes* (2.41%) and *Pseudomonas entomophila* (2.37%). Among the most abundant *Aeromonas* and *Acinetobacter* species in the general hospital influent were *Acinetobacter johnsonii* (9.30%) and *Aeromonas caviae* (4.81%). Notably, the abundances of *Staphylococcus epidermidis* (E.RW 0.27%, E.TW 1.13%) and *Stenotrophomonas maltophilia* (E.RW 0.37%, E.TW 0.82%) increased instead of decreased under the wastewater treatment process of the eye hospital.

### Abundance and Diversity of Antibiotic Resistance Genes

#### Antibiotic Resistance Gene Types

**FIGURE 4:**
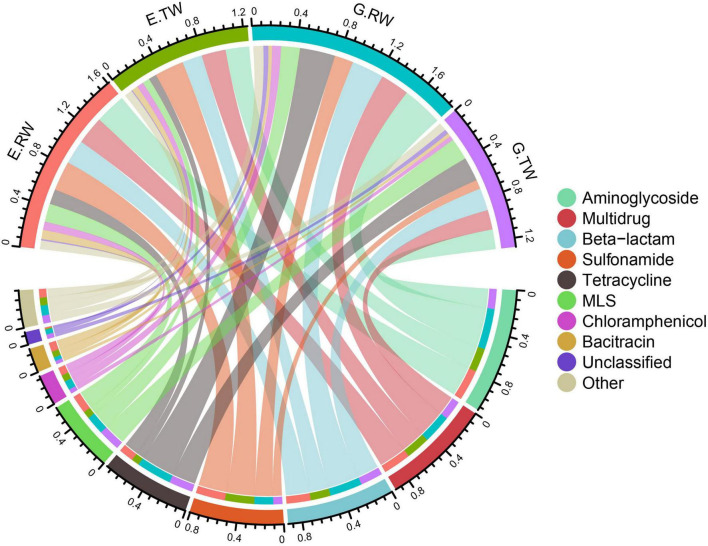
Total ARGs in the four sampling locations. A total of 20 ARG types were identified in 12 samples of hospital wastewater, with the 10 most abundant ARGs shown in the Circos plot. The top half of the plot depicts the abundance of the samples, the bottom half is the abundance of the 10 ARGs, the connecting lines indicate the composition of ARGs in each sample and the distribution of samples in each ARG types.

A total of 20 ARG types were identified in the 12 influent and effluent samples from the two hospitals, and the 10 most abundant ARGs are shown in Figure 4. The total abundance of all ARG types in all the samples ranged from 1.055341 to 2.262843 copies of ARG/copy of the 16S rRNA gene, and aminoglycoside (0.10 ARG/16S rRNA), multidrug (0.092 ARG/16S rRNA) and beta-lactam (0.074 ARG/16S rRNA) resistance genes had the highest abundances, followed by sulfonamide, tetracycline, and macrolide-lincosamide-streptogramin (MLS) resistance genes.

**FIGURE 5:**
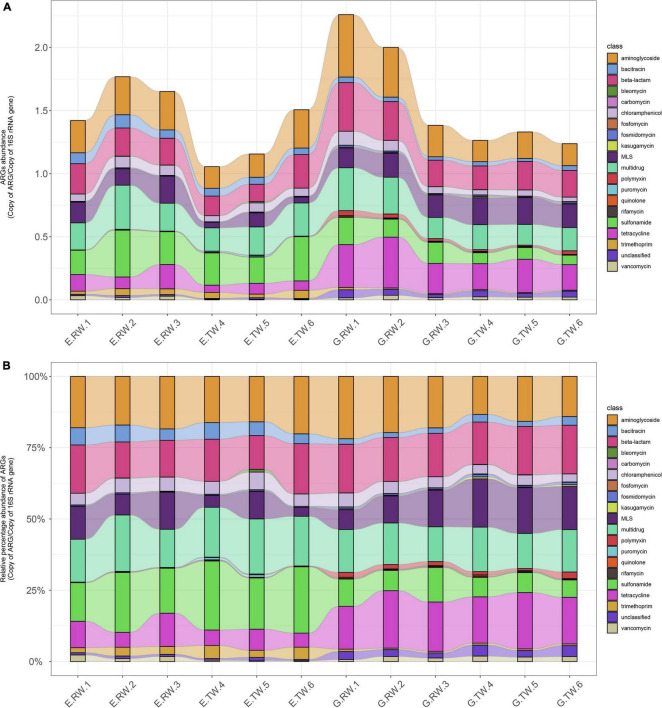
ARGs abundance in each sample. **(A)** Abundance of ARG types in each sample (ARGs copies per copy of 16S rRNA gene). **(B)** Relative percentage of the ARG types in each sample with the total abundance normalized to 100%.

Figure 5A shows the abundances of ARG types in each sample. The average total abundance of ARGs in the influent of eye hospital and general hospital was 1.614 ± 0.177 ARG/16S rRNA and 1.883 ± 0.451 ARG/16S rRNA, respectively, while in the effluent, it was 1.240 ± 0.237 ARG/16S rRNA and 1.278 ± 0.048 ARG/16S rRNA, respectively, indicating that the average total abundance of ARGs in the general hospital influent was higher than that in the eye hospital influent. Moreover, the abundance of ARG detected in the influent of the two hospitals was higher than that in their respective effluent, indicating that both WWTPs effectively reduced the ARG abundances in the wastewater.

Figure 5B shows the relative percent abundance of ARG types in each sample, and the main ARG types and abundance in the two hospitals were different. The most abundant ARG types in the eye hospital influent were aminoglycoside (0.287ARG/16S rRNA), sulfonamide (0.274 ARG/16S rRNA), multidrug (0.262 ARG/16S rRNA), and beta-lactam (0.226 ARG/16S rRNA) resistance genes, which accounted for 64.99% of the total ARGs in the eye hospital influent. Similarly, the most abundant ARG types in the general hospital influent were aminoglycoside (0.379 ARG/16S rRNA), tetracycline (0.327 ARG/16S rRNA) beta-lactam (0.301 ARG/16S rRNA) and multidrug (0.267 ARG/16S rRNA) resistance genes, which accounted for 67.78% of the total ARGs in the general hospital influent. Furthermore, statistical analysis by STAMP (Supplementary Figure 1) showed that there were significant differences in 7 of the 20 ARG types (*P* < 0.05), among which bacitracin and trimethoprim resistance genes were abundant in the eye hospital influent, while tetracycline, quinolone, polymyxin, fosfomycin, and kasugamycin resistance genes were abundant in the general hospital influent, which further indicates that the abundance of ARG types in the influent of the two hospitals was different.

As shown in Figure 5, the abundance of the main ARG types in the effluent of the two hospitals changed, and the treatment also changed the ARG composition in the wastewater of the two hospitals. The vancomycin resistance gene abundance was significantly decreased in the effluent from the eye hospital by the activated sludge treatment process (*P* < 0.05). Nevertheless, the abundance of aminoglycoside and chloramphenicol resistance genes decreased significantly in the effluent from the general hospital by the direct chlorination process (*P* < 0.05). Interestingly, the abundance of MLS resistance genes in the effluent of the general hospital was significantly higher than that in the influent (*P* < 0.05). In other words, the relative abundance of MLS resistance genes increased after treatment.

#### Antibiotic Resistance Gene Subtypes

A total of 715 subtypes were identified in the wastewater of the two hospitals, 463 and 614 subtypes were detected in the influent of the eye hospital and general hospital, respectively, indicating that the ARG subtypes in the influent from the general hospital were more diverse than those from the eye hospital (Supplementary Figure 2). Further analysis of the influent and effluent of the two hospitals showed that the diversity and abundance of ARG subtypes in the eye hospital effluent were significantly lower than those in the influent, indicating that the activated sludge wastewater treatment process reduced the diversity and abundance of ARGs.

**FIGURE 6:**
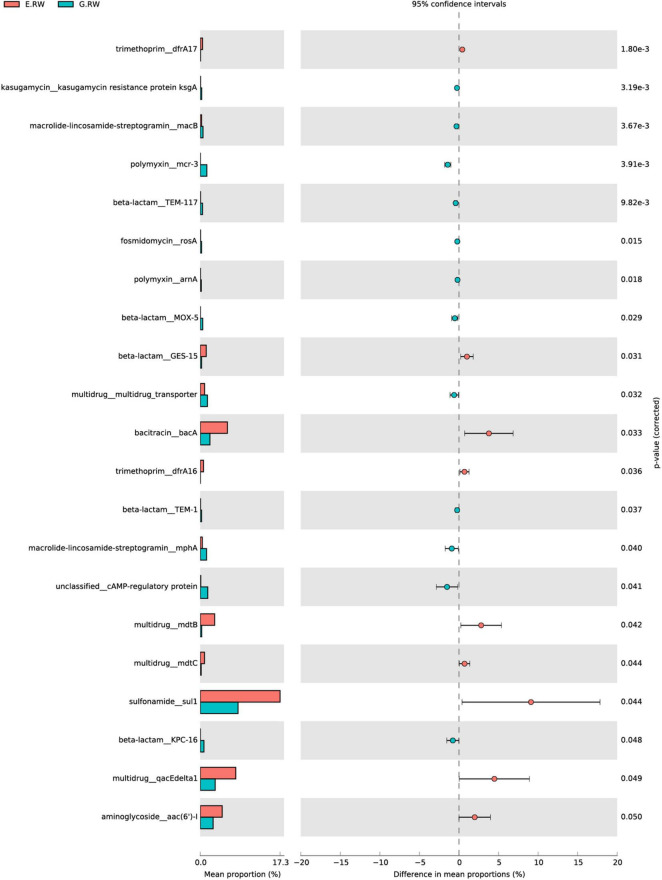
Mean proportion and their differences for discriminative ARG subtypes between samples of E.RW and G.RW.

As shown in Supplementary Figure 3, the influents of the two hospitals contained different abundances of ARG subtypes. The most abundant ARG subtypes in the eye hospital influent were *sul1* (sulfonamides, 0.256 ARG/16S rRNA), *aadA* (aminoglycosides, 0.127 ARG/16S rRNA), *qacE*Δ*1* (MDR, 0.114 ARG/16S rRNA), and *bacA* (*bacteriocins*, 0.0853 ARG/16S rRNA), and these ARG subtypes accounted for 36.8% of the total ARG subtype abundance detected in the eye hospital influent. However, the most abundant ARG subtypes in the general hospital influent were tet39 (tetracyclines, 0.144 ARG/16S rRNA), *sul1* (sulfonamides, 0.129 ARG/16S rRNA), *aph(3″)-I* (aminoglycosides, 0.083 ARG/16S rRNA), and *aadA* (aminoglycosides, 0.071 ARG/16S rRNA), and these ARG subtypes accounted for 22.72% of the total ARG subtype abundance detected in the general hospital influent. Furthermore, the statistical analysis by STAMP showed that 9 subtypes had significantly higher abundances in the eye hospital than in the general hospital, and 12 subtypes had significantly lower abundances in the eye hospital than in the general hospital (Figure 6).

The analysis of the influent and effluent from the two hospitals revealed that the total abundance of ARG subtypes was significantly decreased after treatment by the activated sludge process, and the abundance of most ARG subtypes decreased to a certain extent, of which *lnuB* (MLS), *ermG* (MLS), *aadE* (aminoglycosides), and *mefA* (MLS) showed a significant decrease in abundance (*P* < 0.05). Surprisingly, three of these four subtypes were MLS resistance genes, and the abundance of the tetracycline resistance gene *tetE* did not decrease but increased (*P* < 0.05). We hypothesized that the activated sludge treatment processhad a better removal effect on MLS resistance genes. After the direct chlorination disinfection process in the general hospital, the abundances of most ARG subtypes also decreased to a certain extent, but only one resistance gene, *dfrb1* (trimethoprim), showed a significant decrease (*P* < 0.05). Similar to the activated sludge treatment process, after the direct chlorination disinfection treatment, the abundances of 7 ARG subtypes did not decrease but increased (*P* < 0.05), including 4 tetracycline, 2 beta-lactam, 1 MLS, and 1 vancomycin resistance gene.

#### Antibiotic Resistance Gene Resistance Mechanism Categories

All the ARG subtypes were classified into five primary mechanism categories: 485 antibiotic inactivation, 109 antibiotic efflux, 49 antibiotic target alteration, 29 antibiotic target replacement, and 15 antibiotic target protection. The unclassified mechanism categories were named “others” in this study. The dominant resistance mechanism categories detected in all of the wastewater samples were antibiotic inactivation and antibiotic efflux (Supplementary Figure 4). It is worth noting that each of these wastewaters contained ARGs belonging to all five mechanism categories.

In the eye hospital, the abundance of ARGs in the effluent treated by the activated sludge process was lower than that in the influent, and the abundance of antibiotic target alteration-related genes was significantly lower than that in the influent (*P* < 0.05). In the general hospital using direct chlorination disinfection process, the abundance of antibiotic inactivation-related genes in the effluent was significantly lower than that in the influent (*P* < 0.05), while the abundance of antibiotic target alteration and antibiotic target protection-related genes were higher than that in the influent (*P* < 0.05); the abundances of genes related to other types of resistance mechanisms were also reduced.

### Co-occurrence Patterns Between Antibiotic Resistance Genes and Microbial Taxa

**FIGURE 7:**
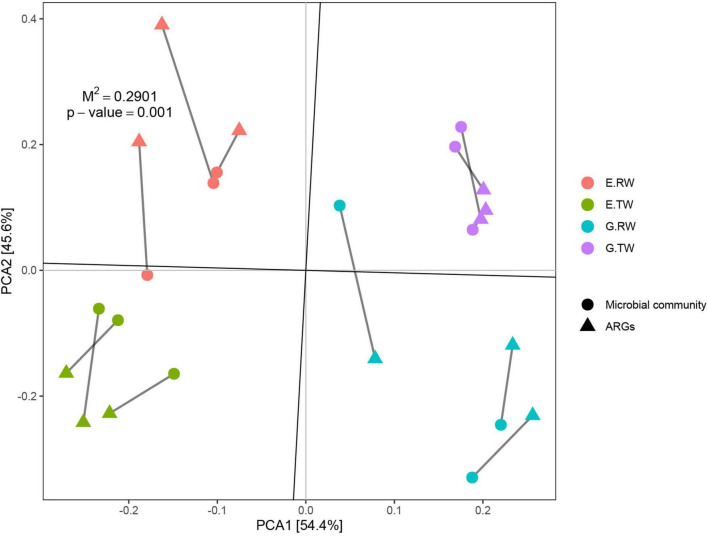
Procrustes analyses of ARGs with microbial community based on the PCA results of ARG subtype and microbial community composition (*M*^2^ = 0.2901, *P* = 0.001, number of permutations = 999).

To study the correlation between ARGs and the microbial community, Procrustes analysis was used to analyze the ARGs and microbial community in these 12 samples, and the results showed that the ARG composition was significantly correlated with the microbial community composition (*M*^2^ = 0.2901, *P* = 0.001). Briefly, changes in the microbial community resulted in different ARG compositions in wastewater (Figure 7). However, procrustes analysis could provide only the overall correlation between ARGs and the microbial community. Therefore, correlation network analysis was performed to identify detailed relationships; this analysis was performed based on the significant correlations (Spearman’s correlation coefficient *R*^2^ > 0.8; *P*-value < 0.01).

**FIGURE 8:**
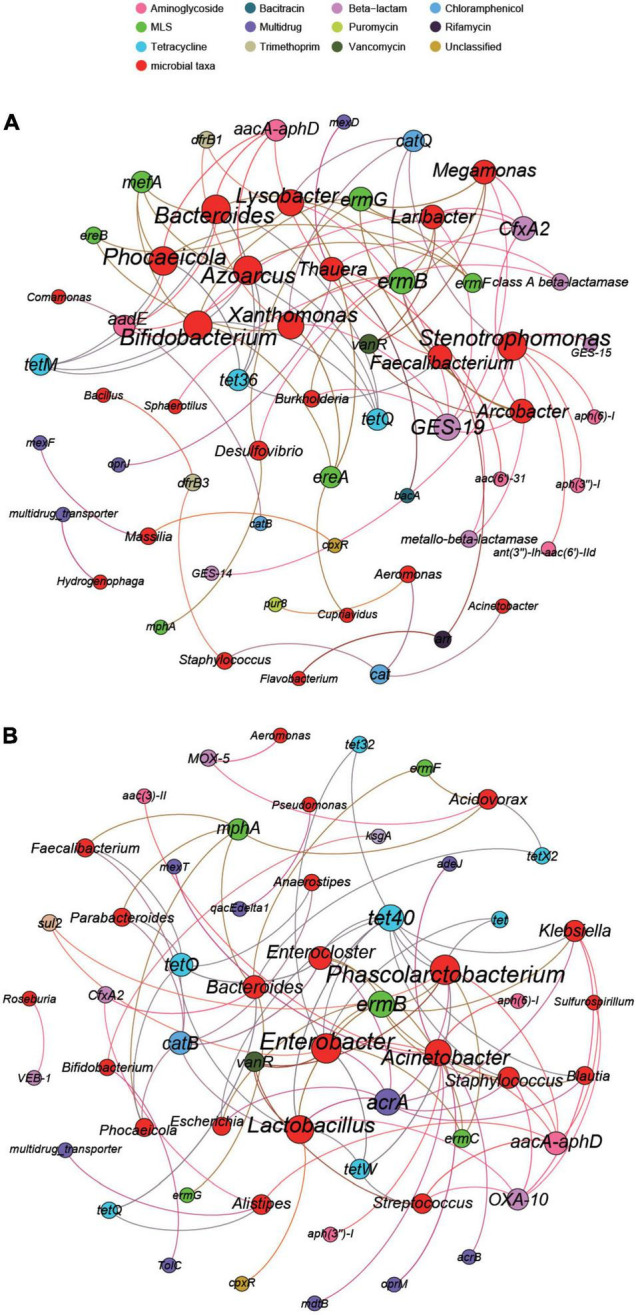
The network analysis of eye hospital wastewater **(A)** and general hospital wastewater **(B)** revealing the co-occurrence patterns between ARG subtypes and microbial taxa. The nodes were colored according to ARG subtypes and microbial taxa. A connection represents a strong (Spearman’s correlation coefficient *R*^2^ > 0.8) and significant (*P*-value < 0.01) correlation. Edges weighted according to the correlation coefficient and node size weighted according to the number of connections.

We selected 30 genera and 70 subtypes with high abundances in the two hospitals for network analysis. The results showed significant differences in the potential primary bacteria carrying ARGs in the wastewater of the two hospitals. As shown in Figure 8A, according to the co-occurrence results, 24 bacterial genera were potential hosts for 36 ARG subtypes in the eye hospital. Among them, *Bifidobacterium* and *Phocaeicola* were the main bacterial genera related to ARGs, serving as potential hosts of 7 ARG subtypes. *Stenotrophomonas* and *Azoarcus* also carry 7 ARG subtypes, but the abundances of these two genera were relatively low. *Xanthomonas* and *Lysobacter* may carry 6 ARG subtypes. However, as shown in Figure 8B, according to the co-occurrence analysis results, 22 bacterial genera were potential hosts for 35 ARG subtypes in the general hospital. Among them, *Enterobacter* and *Phascolarctobacterium* were the main bacterial genera related to ARGs, serving as potential hosts of 10 ARG subtypes; in addition, *Lactobacillus* carries 9 ARG subtypes.

*Bifidobacterium* was the potential host of the majority of the ARGs in the eye hospital, including MLS (*ereA*, *ermG*, *mefA*), tetracycline (*tetM*, *tetQ*), aminoglycoside (*aadE*), and beta-lactam (*CfxA2*) resistance genes; however, *Bifidobacterium* was a potential host carrying only two ARG subtypes, kasugamycin (kasugamycin resistance protein *KsgA*), MDR (*TolC*), in the general hospital wastewater. In the general hospital, *Acinetobacter* was the potential host of the majority of the ARGs, including MDR (*acrB*, *adeJ*, *mdtB*, *mexT*), aminoglycoside [*aac(3)-II*, *aph(3″)-I*, *aph(6)-I*], and MLS (*oprM*) resistance genes, but in eye hospital wastewater, *Acinetobacter* was a potential host carrying only one ARG subtype, namely, chloramphenicol (*cat*). In conclusion, the same bacteria in the eye hospitals and the general hospitals may carry different ARG subtypes, and the same ARG subtype may also be carried by different bacteria in the two types of hospitals.

## Discussion

Potential pathogenic bacteria and ARGs in hospital wastewater pose a considerable threat to public health. Metagenomic analysis revealed the complexity of the microbial communities and provided a functional profile of ARGs and patterns of co-occurrence between ARGs and potential host bacteria (Chakraborty et al., 2020). Our study identified the distribution and prevalence of bacterial community and ARGs in the Eye and General hospital wastewater and attempted to evaluate the effects of the activated sludge treatment process and the direct chlorination disinfection treatment process in hospital wastewater. Studies on different types of hospitals before and after wastewater treatment offer an objective basis for recommending appropriate treatment methods for certain clinical environments.

In this study, we found that the microbial community structure in the influent was different between the two types of hospitals, and there were many clinically pathogenic bacteria present in the samples. Previous studies on hospital WWTPs (HWWTPs) and urban WWTPs (UWWTPs) have also shown that *Proteobacteria*, *Bacteroides*, *Firmicutes*, and *Actinobacteria* dominate the influent (Tang et al., 2016; Lira et al., 2020; Manoharan et al., 2021; Rodriguez et al., 2021). The human gut microbes predominant in influent may originate from residues of human feces released into wastewater and may even include pathogens from patients (Karkman et al., 2019). Wastewater is rich in nitrogen, phosphorus, and other nutrients, which promote the growth and reproduction of bacteria. *Proteobacteria* and *Firmicutes* include several of the most important human bacterial pathogens, such as *Escherichia coli*, *Salmonella*, *Pseudomonas aeruginosa*, *Klebsiella pneumoniae*, and *Staphylococcus aureus*. *Bacteroides* participate in many important metabolic activities in the human colon. Actinomycetes play an important role in the decomposition of organic matter. At the genus level, *Thauera* is abundant in activated sludge and can grow in aerobic and denitrifying conditions with large quantities of organic matter and aromatic compounds (Mechichi et al., 2002). *Pseudomonas*, *Aeromonas*, and *Acinetobacter* include most of the common clinical pathogens, such as *Pseudomonas aeruginosa* and *Acinetobacter baumannii* often cause urinary tract infections and meningitis, etc. (Girija et al., 2020; Motbainor et al., 2020; Kizilates et al., 2021). These are also the key pathogens associated with hospital and community-acquired infections (Dijkshoorn et al., 2007). Varela et al. (2016) found that *Aeromonas* contained various ARGs in wastewater and suggested that this genus can be used as a tracer for monitoring the spread of antibiotic resistance in wastewater. In summary, hospital wastewater contains a high proportion of potential pathogens, and research on the potential microbiota of hospital wastewater and on the clinical pathogens present is particularly important.

The abundance and diversity of ARGs in the general hospital were higher than those in the eye hospital, and differences in human medical activities play an important role. The ARG types with high abundance correspond to the types of antibiotics commonly used in hospital medical treatment (Cai et al., 2021). Although in the same hospital different medical activities (surgical source wastewater and patient living source wastewater) have found that there were differences in the microbial community and ARGs (Manoharan et al., 2021). Patients in general hospitals and eye hospitals widely use different types of antibiotics, promoting and maintaining the occurrence of various ARGs (Cai et al., 2021). Many studies have shown that hospital wastewater contains a high proportion of clinical pathogens, and general hospitals have a more complex clinical pathogen spectrum than eye hospitals. The larger scale of general hospitals than eye hospitals, accommodating more patients and bearing greater pressure, which is one of the reasons why microbial communities in general hospitals are more diverse (Lamba et al., 2017).

The ARG abundances in the influents of the general hospital were significantly higher than eye hospital influents, and the ARG types may be closely related to the hospital types. Aminoglycoside resistance genes were the most abundant in the influent of the two hospitals. In addition, greater abundances of sulfonamide resistance genes were found in the eye hospital, while greater abundances of tetracycline resistance genes were found in the general hospital. This result is consistent with the findings of Guo et al. (2021). Their study found that tetracycline resistance genes were the most abundant in untreated wastewater in a general hospital, while bacitracin resistance genes were the more abundant in untreated wastewater in a stomatology hospital. Based on these findings, we speculate that the main types of ARGs in the wastewater of specialist hospitals are different from those of general hospitals, and the ARG types also differ among different types of specialist hospitals. Tetracycline resistance genes generally have a higher abundance in the wastewater of general hospitals. This may be because tetracycline has been widely used to treat humans and tetracycline resistance genes are ubiquitous and abundant in the human gut (Hu et al., 2013). At present, tetracycline drugs are used cautiously to treat human diseases.

Pathogenic bacteria carrying ARGs pose a higher risk to human health (Shi et al., 2021). Multidrug-resistant bacteria detected in hospital wastewater pose a considerable threat to public health. *Bifidobacterium* has been recommended as a potential indicator of human fecal contamination in surface water, and its high correlation with ARGs also highlights the impact of human feces and human intestinal flora carrying ARGs on the spread of ARGs (Nebra et al., 2003). *Enterobacter* is considered to be a repository of ARGs (Diene and Rolain, 2013). Among these bacteria, HGT of *Klebsiella pneumoniae* resistance genes has been considered to be the main reason for the widespread existence of MDR in the hospital environment. Studies have shown that *Acinetobacter* can multiply in the hospital environment and cause up to 9% of hospital infections (Joly-Guillou, 2005). The differences between our results and those of previous studies provide a reference for predicting other new potential ARG hosts, and the corresponding relationship needs to be further verified by other methods, such as binning.

In addition, we found different changes in the effluent of the two types of hospitals. The uniformity and richness of the microbial community in the wastewater changed significantly, and the effects of different treatment methods were also different. The species diversity increased significantly after the activated sludge wastewater treatment, while the direct chlorination disinfection process did not increase the diversity. Increased species diversity in the effluent was also found in wastewater samples from other HWWTPs or UWWTPs (Lira et al., 2020; Rodriguez-Mozaz et al., 2020; Manoharan et al., 2021). Changes in alpha diversity and the appearance of several genera belonging to specific microbial communities may be related to the process conditions of individual treatments (Hu et al., 2012; Wolff et al., 2018). The activated sludge process uses sludge-like flocs formed by the reproduction of aerobic microorganisms. This floc has a strong ability to adsorb and oxidize organic matter. Studies have shown that aerobic activated sludge samples have high species richness (Johnson et al., 2015), and chlorination achieves the disinfection effect directly through oxidation. In addition, the increase in species diversity during wastewater treatment may be related to the formation of biofilms in the WWTP environment (Wakelin et al., 2008; Peng et al., 2018). However, considering that there are many possible reasons for the differences in microbial community structure, more comprehensive and in-depth studies are needed to verify this hypothesis.

Similarly, the ARGs in the two types of hospitals effluent also changed in significantly different manners. *Sul1* can be transferred between bacteria in different environments, and its high abundance indicates that the potential risk of HGT between different bacteria in the environment is high (He et al., 2020). The treatment process of the WWTPs was effective in removing part of the ARGs (Tang et al., 2016; Li et al., 2018; Pallares-Vega et al., 2019; Shi et al., 2021), but it also promoted bacterial growth and ARG exchange, leading to a further increase in ARGs (Du et al., 2015; Farkas et al., 2016; Kumar et al., 2020). Whether wastewater is safe after treatment has always been the focus of debate. Previous research results have shown that the effectiveness of the chlorination process to eliminate ARGs is limited (Auerbach et al., 2007; Munir et al., 2011), and the abundance of ARB increases after chlorination, including of bacteria belonging to *Enterobacteriaceae* (Mokracka et al., 2012; Al-Jassim et al., 2015). In this study, we found that the direct chlorination- disinfection process had a poor removal effect on MLS resistance genes and even enriched these genes. In contrast, the activated sludge treatment had a better effect on the removal of MLS resistance genes. Moreover, we found that the activated sludge wastewater treatment has a better effect on the elimination of ARGs than the direct chlorination disinfection. Only a *tetE* (tetracycline) increased after treatment and our current medical needs for tetracycline antibiotics are low. However, some researchers believe that the effects of direct chlorination and the activated sludge process are equivalent, even though the activated sludge treatment process takes significantly longer than the direct chlorination disinfection process (Azuma and Hayashi, 2021).

The above results suggest that we should pay more attention to the treatment and emission standards for hospital wastewater. According to the specific situation of hospital wastewater, more reasonable and effective strategies should be formulated and implemented to limit the increase and spread of antibiotic resistance in the environment. Increasing the sampling locations and the number of samples sequenced will be helpful to monitor the microbial trends and diversity shifts. Linking wastewater strains with hospital clinical isolates to analyze the potential correlation between the ARB and ARG should also be considered in future hospital wastewater studies.

In conclusion, we used high-throughput metagenomic sequencing technology to analyze the bacterial community composition and the prevalence of ARGs before and after wastewater treatment in the two hospitals exposed to different antibiotic usage conditions (an eye hospital and a general hospital). We found that there were significant differences in the bacterial community and ARG composition of the influent between the two hospitals. The same bacteria in wastewater from different hospitals could carry different ARGs, and the same ARG could also be carried by different bacteria. The activated sludge treatment process and the direct chlorination disinfection have different effects on bacterial communities and ARGs in wastewater but also have limitations. We found that the activated sludge wastewater treatment has a better effect on the elimination of ARGs than the direct chlorination disinfection. Therefore, it is crucial to select appropriate wastewater treatment methods according to the specific wastewater conditions to reduce the wide distribution of ARB and ARGs in the water environment.

## Data Availability Statement

The datasets presented in this study can be found in online repositories. The names of the repository/repositories and accession number(s) can be found below: https://www.ncbi.nlm.nih.gov/, PRJNA770854.

## Author Contributions

MZ and YLo contributed to the conception, design, and interpretation of the data. XM and XD contributed to data analysis and drafted the manuscript. JC, CF, JY, YLi, YZ, TW, and SL contributed to sample collection and DNA extraction. All authors approved the final version of the manuscript.

## Conflict of Interest

The authors declare that the research was conducted in the absence of any commercial or financial relationships that could be construed as a potential conflict of interest.

## Publisher’s Note

All claims expressed in this article are solely those of the authors and do not necessarily represent those of their affiliated organizations, or those of the publisher, the editors and the reviewers. Any product that may be evaluated in this article, or claim that may be made by its manufacturer, is not guaranteed or endorsed by the publisher.

## Footnotes

1
http://huttenhower.sph.harvard.edu/kneaddata

2
http://www.r-project.org/

## Supplementary Material

Supplementary Figure 1 Mean proportion and their differences for discriminative ARG types between samples of E.RW and G.RW.

Supplementary Figure 2 Numbers of shared ARG subtypes by E.RW, E.TW, G.RW, and G.TW samples.

Supplementary Figure 3 Abundance of the 100 major ARG subtypes in the samples.

Supplementary Figure 4 The abundance of ARGs belonging to different resistance mechanism categories.
