# Supplementary figures and images for: Metagenomic Analysis Reveals Changes in Bacterial Communities and Antibiotic Resistance Genes in an Eye Specialty Hospital and a General Hospital Before and After Wastewater Treatment
Source: Front Microbiol. 2022 May 19;13:848167. doi: 10.3389/fmicb.2022.848167 (PMC9162037; doi:10.3389/fmicb.2022.848167)

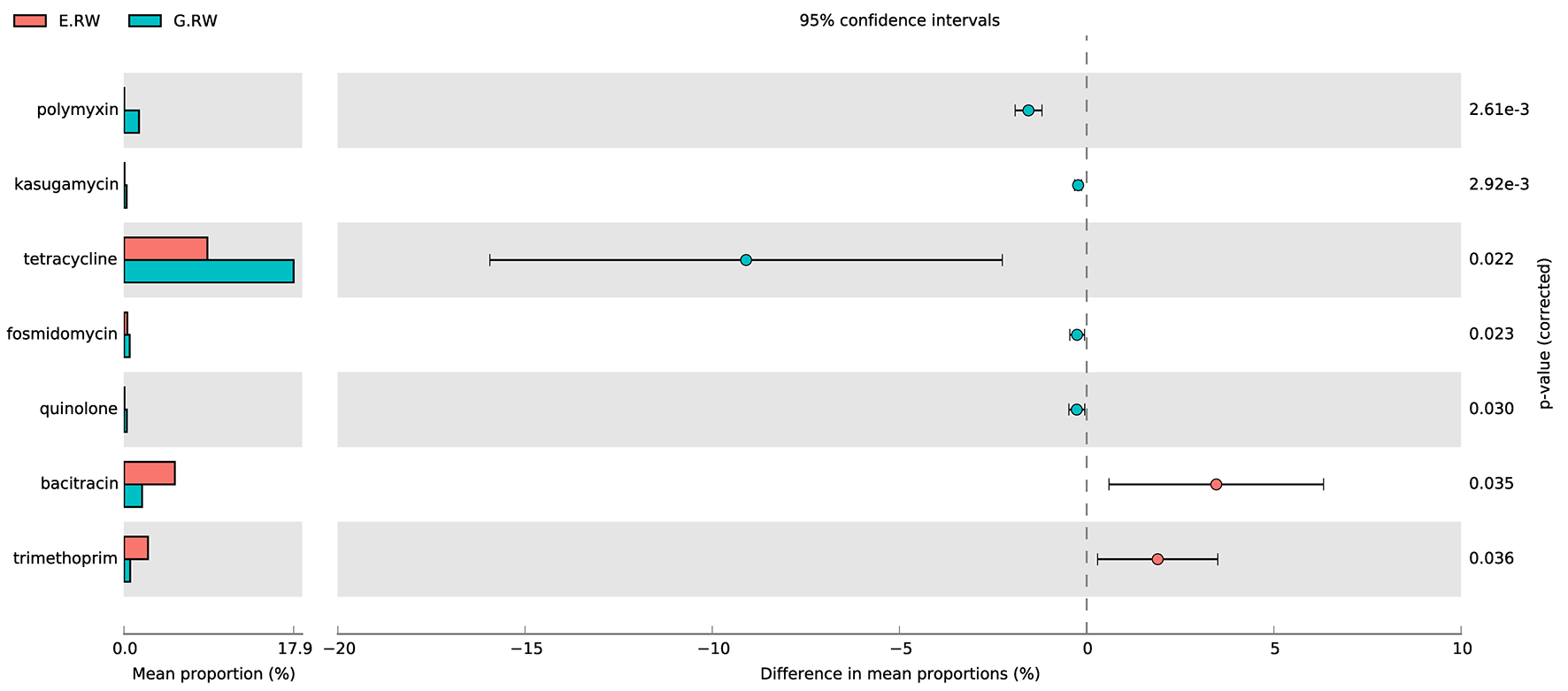

Supplement: Supplementary Figure 1 — Mean proportion and their differences for discriminative ARG types between samples of E.RW and G.RW. [file Image_1.TIF]

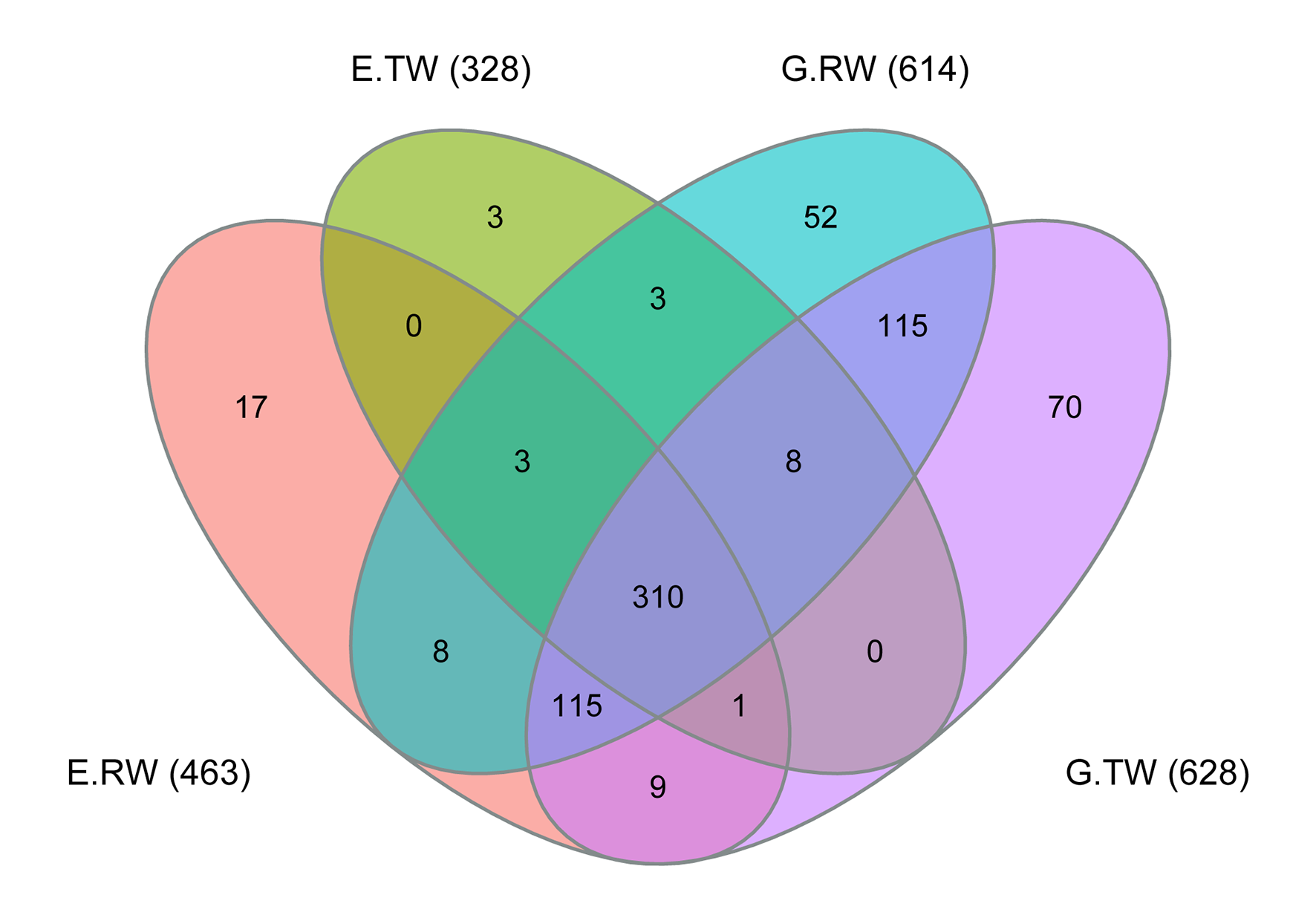

Supplement: Supplementary Figure 2 — Numbers of shared ARG subtypes by E.RW, E.TW, G.RW, and G.TW samples. [file Image_2.TIF]

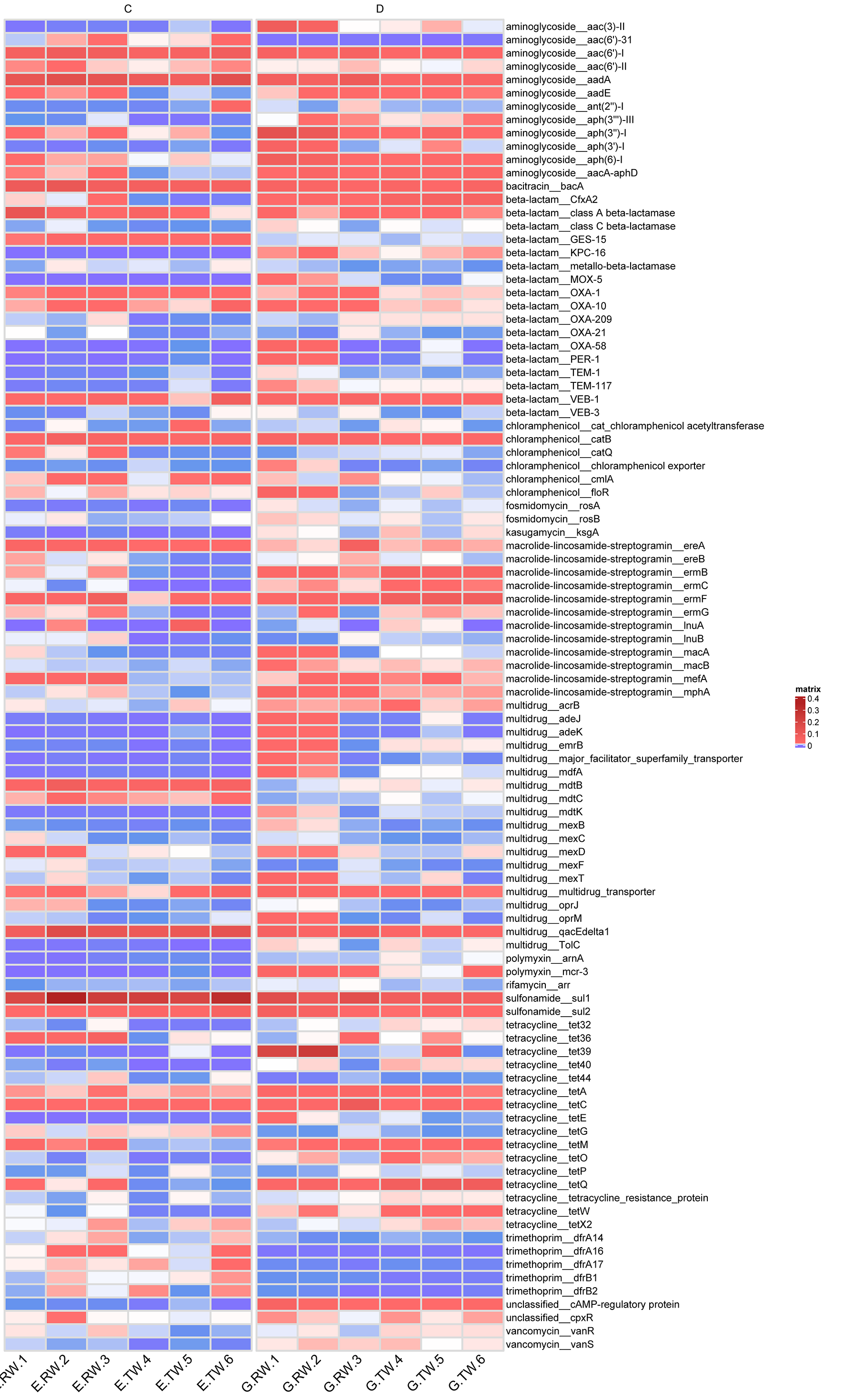

Supplement: Supplementary Figure 3 — Abundance of the 100 major ARG subtypes in the samples. [file Image_3.TIF]

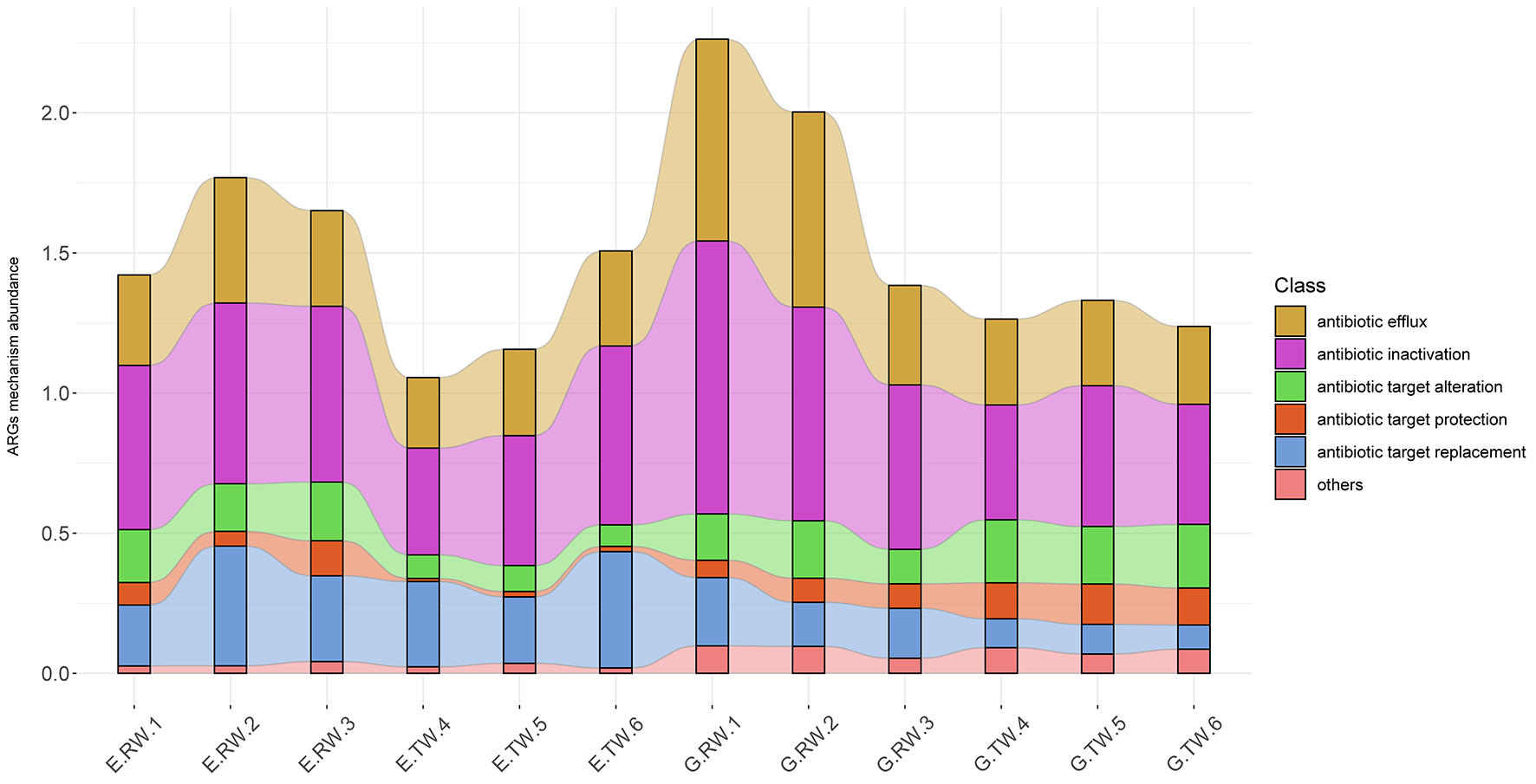

Supplement: Supplementary Figure 4 — The abundance of ARGs belonging to different resistance mechanism categories. [file Image_4.TIF]
